# Evolution and Diversity of *Listeria monocytogenes* from Clinical and Food Samples in Shanghai, China

**DOI:** 10.3389/fmicb.2016.01138

**Published:** 2016-07-22

**Authors:** Jianmin Zhang, Guojie Cao, Xuebin Xu, Marc Allard, Peng Li, Eric Brown, Xiaowei Yang, Haijian Pan, Jianghong Meng

**Affiliations:** ^1^National and Regional Joint Engineering Laboratory for Medicament of Zoonosis Prevention and Control, Key Laboratory of Zoonosis Prevention and Control of Guangdong Province, College of Veterinary Medicine, South China Agricultural UniversityGuangzhou, China; ^2^Department of Nutrition and Food Science and Joint Institute for Food Safety and Applied Nutrition, University of Maryland, College ParkCollege Park, MD, USA; ^3^Shanghai Municipal Center for Disease Control and PreventionShanghai, China; ^4^Division of Microbiology, Office of Regulatory Science, Center for Food Safety and Applied Nutrition, U.S. Food and Drug AdministrationCollege Park, MD, USA; ^5^Institute of Disease Control and Prevention, Academy of Military Medical ScienceBeijing, China; ^6^Department of Food Science & Technology, School of Agriculture and Biology, Shanghai Jiao Tong UniversityShanghai, China

**Keywords:** *L. monocytogenes*, evolution, whole genome analysis, plasmid, China

## Abstract

*Listeria monocytogenes* is a significant foodborne pathogen causing severe systemic infections in humans with high mortality rates. The objectives of this work were to establish a phylogenetic framework of *L. monocytogenes* from China and to investigate sequence diversity among different serotypes. We selected 17 *L. monocytogenes* strains recovered from patients and foods in China representing serotypes 1/2a, 1/2b, and 1/2c. Draft genome sequences were determined using Illumina MiSeq technique and associated protocols. Open reading frames were assigned using prokaryotic genome annotation pipeline by NCBI. Twenty-four published genomes were included for comparative genomic and phylogenetic analysis. More than 154,000 single nucleotide polymorphisms (SNPs) were identified from multiple genome alignment and used to reconstruct maximum likelihood phylogenetic tree. The 41 genomes were differentiated into lineages I and II, which consisted of 4 and 11 subgroups, respectively. A clinical strain from China (SHL009) contained significant SNP differences compared to the rest genomes, whereas clinical strain SHL001 shared most recent common ancestor with strain SHL017 from food. Moreover, clinical strains SHL004 and SHL015 clustered together with two strains (08-5578 and 08-5923) recovered from an outbreak in Canada. Partial sequences of a plasmid found in the Canadian strain were also present in SHL004. We investigated the presence of various genes and gene clusters associated with virulence and subgroup-specific genes, including internalins, *L. monocytogenes* pathogenicity islands (LIPIs), *L. monocytogenes* genomic islands (LGIs), stress survival islet 1 (SSI-1), and clustered regularly interspaced short palindromic repeats (CRISPR)/cas system. A novel genomic island, denoted as LGI-2 was identified. Comparative sequence analysis revealed differences among the *L. monocytogenes* strains related to virulence, survival abilities, and attributes against foreign genetic elements. *L. monocytogenes* from China were genetically diverse. Strains from clinical specimens and food related closely suggesting foodborne transmission of human listeriosis.

## Introduction

*Listeria monocytogenes* causes severe systemic infections with high mortality rates (Toledo-Arana et al., 2009; Kuenne et al., 2013) and has been responsible for numerous outbreaks in North America and Europe during recent years (Gilmour et al., 2010; Jackson et al., 2011; Smith et al., 2011; Laksanalamai et al., 2012; Schoder et al., 2014). *L. monocytogenes* is able to survive and grow under a wide range of temperature and pH conditions with a significant tolerance to salt (Gilmour et al., 2010). This ubiquitous pathogen imposes a risk with significant economic burden to public health (Toledo-Arana et al., 2009).

*L. monocytogenes* consists of four evolutionary lineages and 13 identified serotypes, with serotypes 1/2a, 1/2b, 1/2c, and 4b causing most human listeriosis cases (Swaminathan and Gerner-Smidt, 2007; Ragon et al., 2008; den Bakker et al., 2013). Serotypes 1/2b and 4b belong to lineage I; 1/2a and 1/2c belong to lineage II (Ragon et al., 2008). Serotypes 1/2a, 1/2b, and 1/2c accounted for more than 90% of *L. monocytogenes* isolated from food in China (Wang et al., 2012).

Central to *L. monocytogenes* pathogenesis is the ability to invade and cross host barriers (Bergmann et al., 2013) and to secrete proteins beyond the cell surface using internalins and various secretion systems (Desvaux and Hebraud, 2006). The internalin family is composed of important proteins involving virulence activities of *L. monocytogenes*. Internalins A (InlA) and B (InlB) played essential roles in invasion activities (Dussurget et al., 2004). Several additional internalins InlJ, InlI, and InlK have been identified recently (Sabet et al., 2005; Neves et al., 2013; Becavin et al., 2014). There are six protein secretion systems in *L. monocytogenes*, including Sec (secretion system), Tat (twin-arginine translocation), FPE (fimbriae protein exporter), FEA (flagella export apparatus), holins, and Wss (WXG100 secretion system) (Desvaux and Hebraud, 2006).

Similar to *Salmonella* (Cao et al., 2013) and *Escherichia coli* (Ju et al., 2014), *L. monocytogenes* contains genomic islands playing important roles in virulence, such as *Listeria* pathogenicity islands (LIPIs) (Gonzalez-Zorn et al., 2000; Clayton et al., 2014) and *Listeria* genomic islands (LGIs) (Gilmour et al., 2010). LIPI-1 contains virulence determinants, like *hly, plcAB*, and *actA* (Gonzalez-Zorn et al., 2000). Moreover, *L. monocytogenes* carries a gene cluster termed stress survival islet 1 (SSI-1), which is composed of five genes contributing to the survival of cells in suboptimal conditions of food environments such as low pH and high salt concentrations (Ryan et al., 2010).

Clustered regularly interspaced short palindromic repeats (CRISPR)/cas system is considered a bacterial immune system against invading genetic fragments by targeting specific sequence including phages and plasmids (Touchon and Rocha, 2010). The presence of functional CRISPR/cas system has a negative correlation with resistance to antibiotics in *Enterococci* (Palmer and Gilmore, 2010). In addition, non-coding RNAs regulate gene expression through hybridizing with mRNA or binding to proteins to modulate their activities under different conditions (Kuenne et al., 2013). Both of these systems contribute to the pathogenicity, antimicrobial resistance, and metabolism of *L. monocytogenes*.

The objectives of the current study were to provide a phylogenetic framework of human and foodborne *L. monocytogenes* from China, and to reveal genomic sequence diversities of *L. monocytogenes*. The data should assist in a better understanding of the evolution and genetic diversity of *L. monocytogenes*.

## Materials and methods

### Bacterial strains

To determine the genetic diversity and to identify the genetic characteristics of *L. monocytogenes*, 12 clinical strains belonging to different PFGE profiles were selected among 50 strains from Shanghai, China (2004–2012) (unpublished data) (Table 1). Moreover, five strains of *L. monocytogenes* from food were included to investigate a possible connection of food to the clinical cases. The strains represented serotypes 1/2a (*n* = 8), 1/2b (*n* = 5), and 1/2c (*n* = 4).

**Table 1:** **Metadata Associated with 17 *L. monocytogenes* strains from Shanghai, China**.

**Strain**	**Serotype**	**Lineage**	**Year**	**Sources**	**Contigs**	**N50 size**	**STs**
SHL001	1/2a	II	2007	Human#	33	476,844	381
SHL004	1/2a	II	2008	Human	18	579,300	8
SHL005	1/2a	II	2008	Human	17	437,049	7
SHL009	1/2a	II	2012	Human^*^	32	541,739	91
SHL011	1/2a	II	2011	Human	17	543,519	29
SHL013	1/2a	II	2012	Human^*^	20	358,858	391
SHL002	1/2b	I	2007	Human	22	476,844	3
SHL007	1/2b	I	2011	Human	43	355,359	87
SHL008	1/2b	I	2012	Human	26	293,078	3
SHL010	1/2b	I	2012	Human	84	259,950	2
SHL012	1/2b	I	2010	Human#	23	355,398	87
SHL006	1/2c	II	2010	Human	30	476,139	9
SHL015	1/2a	II	2008	Beef	15	425,029	8
SHL017	1/2a	II	2004	Bean	17	726,747	381
SHL014	1/2c	II	2008	Pork	23	477,674	9
SHL016	1/2c	II	2008	Fish	17	512,641	9
SHL018	1/2c	II	2004	Vegetables	18	429,471	9

# *Cerebrospinal fluid; all other clinical strains were isolated from blood*.

* *The host of these strains died*.

### Genome sequencing and annotation

Whole genome sequencing was performed on the 17 strains using Illumina MiSeq technique and associated protocols (Illumina, San Diego, CA) with MiSeq Reagent Kit v2 (500 cycle), Nextera XT DNA Sample Preparation kit, and Nextera XT Index Kit. Draft genome data were assembled using CLC Genomic Workbench *de novo* and were annotated by NCBI using Prokaryotic Genomes Annotation Pipeline (Klimke et al., 2009). These draft genomes were deposited in GenBank under the following accession numbers: SHL001 (APIB00000000), SHL002 (APIB00000000), SHL004 (APIB00000000), SHL005 (APIB00000000), SHL006 (APIB00000000), SHL007 (APIB00000000), SHL008 (APIB00000000), SHL009 (APIB00000000), SHL010 (APIB00000000), SHL011 (APIB00000000), SHL012 (APIB00000000), SHL013 (APIB00000000), SHL014 (AWWQ00000000), SHL015 (AWWR00000000), SHL016 (AWWS00000000), SHL017 (AWWT00000000), and SHL018 (AWWU00000000).

### Genomic analysis

In addition to the 17 genomes, 24 publicly available *L. monocytogenes* genomes were selected to determine the evolutionary relationship among *L. monocytogenes* (Table 2). Single nucleotide polymorphisms (SNPs) were identified based on core genome alignments using progressive Mauve (Darling et al., 2010) and customized in-house script. Clonal complexes were determined by multilocus sequence typing (MLST) analysis using seven housekeeping genes, including *abcZ, bglA, cat, dapE, dat, Idh*, and *IhkA* (Cantinelli et al., 2013). To reconstruct evolutionary relatedness among the genomes, we used Genetic Algorithm for Rapid Likelihood Inference (GARLI 2.0) to perform a maximum likelihood analysis (Zwickl, 2006) with 1000 bootstrap replicates and GTR+I+G nucleotide substitution model based on the SNPs we identified. Pairwise distance matrix with the number of nucleotide differences was calculated using MEGA 5.10 (Tamura et al., 2011) with 10,000 bootstrap replications. CRISPR arrays and *cas* gene clusters were identified using CRISPRFinder (Grissa et al., 2007). Stand-alone blast (blast 2.27+) (Altschul et al., 1990) was used to determine the presence/absence of 117 virulence related genes, 150 non-coding RNAs (Izar et al., 2011), and other gene clusters. Strain specific elements within subgroups were identified using cluster_smallmem command in the USEARCH package (Edgar, 2010) with 90% identities as threshold. The genome organization comparison for subgroup IIk was displayed using BRIG 0.95 with 90 and 70% as upper and lower identity threshold, respectively (Alikhan et al., 2011).

**Table 2:** **Sequencing Statistics for 41 Selected Strains of *L. monocytogenes***.

**Strain**	**Serotype/Lineage**	**Genome Size**	**GC Content**	**CDS**	**Source**	**Year**	**Country**	**GenBank Accession**
SHL001	1/2a, II	2.95	37.9	2964	Human	2007	China	APIB00000000
SHL004	1/2a, II	3.01	37.8	3018	Human	2008	China	APID00000000
SHL005	1/2a, II	2.88	37.9	2859	Human	2008	China	APIE00000000
SHL009	1/2a, II	2.87	37.9	2866	Human	2012	China	APII00000000
SHL011	1/2a, II	2.87	37.9	2847	Human	2011	China	APIK00000000
SHL013	1/2a, II	2.86	37.9	2821	Human	2012	China	APIM00000000
SHL015	1/2a, II	2.96	38.0	2946	Beef	2008	China	AWWR00000000
SHL017	1/2a, II	2.95	38.0	2937	Bean	2004	China	AWWT00000000
SHL002	1/2b, I	3.12	37.9	3113	Human	2007	China	APIC00000000
SHL007	1/2b, I	2.98	37.9	2995	Human	2011	China	APIG00000000
SHL008	1/2b, I	3.01	37.9	2990	Human	2012	China	APIH00000000
SHL010	1/2b, I	3.08	37.9	3112	Human	2012	China	APIJ00000000
SHL012	1/2b, I	2.93	37.9	2906	Human	2010	China	APIL00000000
SHL006	1/2c, II	2.93	37.9	2959	Human	2010	China	APIF00000000
SHL014	1/2c, II	2.95	37.9	2952	Pork	2008	China	AWWQ00000000
SHL016	1/2c, II	2.97	37.7	2992	Fish	2008	China	AWWS00000000
SHL018	1/2c, II	2.94	37.8	2948	Vegetables	2004	China	AWWU00000000
10403S	1/2a, II	2.90	38.0	2814	Human	1968	U.S.	CP002002
F6900	1/2a, II	2.97	37.7	3005	Human	1989	U.S.	AARU02000000
J2-031	1/2a, II	2.96	37.9	2924	Human	1996	U.S.	CP006593
J2818	1/2a, II	2.97	37.7	3083	Human	2000	U.S.	AARX02000000
J0161	1/2a, II	3.00	37.9	2955	Human	2000	U.S.	CP002001
08-5578	1/2a, II	3.03	38.0	3088	Human	2008	Canada	CP001602.1
08-5923	1/2a, II	3.00	38.0	2966	Human	2008	Canada	CP001604
SLCC5850	1/2a, II	2.91	38.0	2866	Rabbit	1924	UK	FR733647
EGD-e	1/2a, II	2.94	38.0	2846	Rabbit	1926	UK	AL591824.1
F6854	1/2a, II	2.95	37.8	2967	Hot dog	1988	U.S.	AADQ01000000
C1-387	1/2a, II	2.99	37.9	2953	Food	1999	U.S.	CP006591
FSL J2-003	1/2a, II	2.74	37.8	2937	N.a.	N.A.	U.S.	AARM02000000
FSL N3-165	1/2a, II	2.88	37.8	2890	Soil	N.A.	U.S.	AARQ02000000
FSL R2-503	1/2b, I	2.99	37.8	3027	Human	1994	U.S.	AARR00000000
SLCC2755	1/2b, I	2.97	38.1	2940	Human	N.A.	N.A.	NC_018587
FSL J1-194	1/2b, I	2.99	37.8	3012	Human	N.A.	U.S.	AARJ00000000
R2-502	1/2b, I	3.03	37.9	2984	Food	1994	U.S.	CP006594
J2-1091	1/2b, I	2.98	37.9	2912	Animal	1995	U.S.	CP006596
FSL J1-175	1/2b, I	2.87	37.9	3147	Water	2006	U.S.	AARK00000000
FSL J2-064	1/2b, I	2.83	37.9	2934	Food	N.A.	N.A.	AARO00000000
N1-011A	1/2b, I	3.01	37.9	3059	Environment	N.A.	U.S.	CP006597
LO28	1/2c, II	2.68	37.8	2999	Human	N.A.	N.A.	AARY00000000
FSL R2-561	1/2c, II	2.97	38.0	2910	N.A.	N.A.	N.A.	NC_017546
SLCC 2372	1/2c, II	2.97	38.0	2990	N.A.	N.A.	N.A.	NC_018588

## Results

Our findings revealed the phylogeny of 41 *L. monocytogenes* strains from diverse sources and geographic locations, and provided insights into their sequence diversities. The size of draft genomes of the 17 strains from Shanghai, China (Table 1) ranged from 2.86 Mb (SHL013) to 3.12 Mb (SHL002) (Table 2). SHL013 contained the lowest number of genes (*n* = 2821) whereas SHL002 had the highest number of genes (*n* = 3113). The average genome size in lineages I and II was 2.93 Mb and 2.98 Mb, respectively.

### Phylogenetic analysis between strains

**Figure 1:**
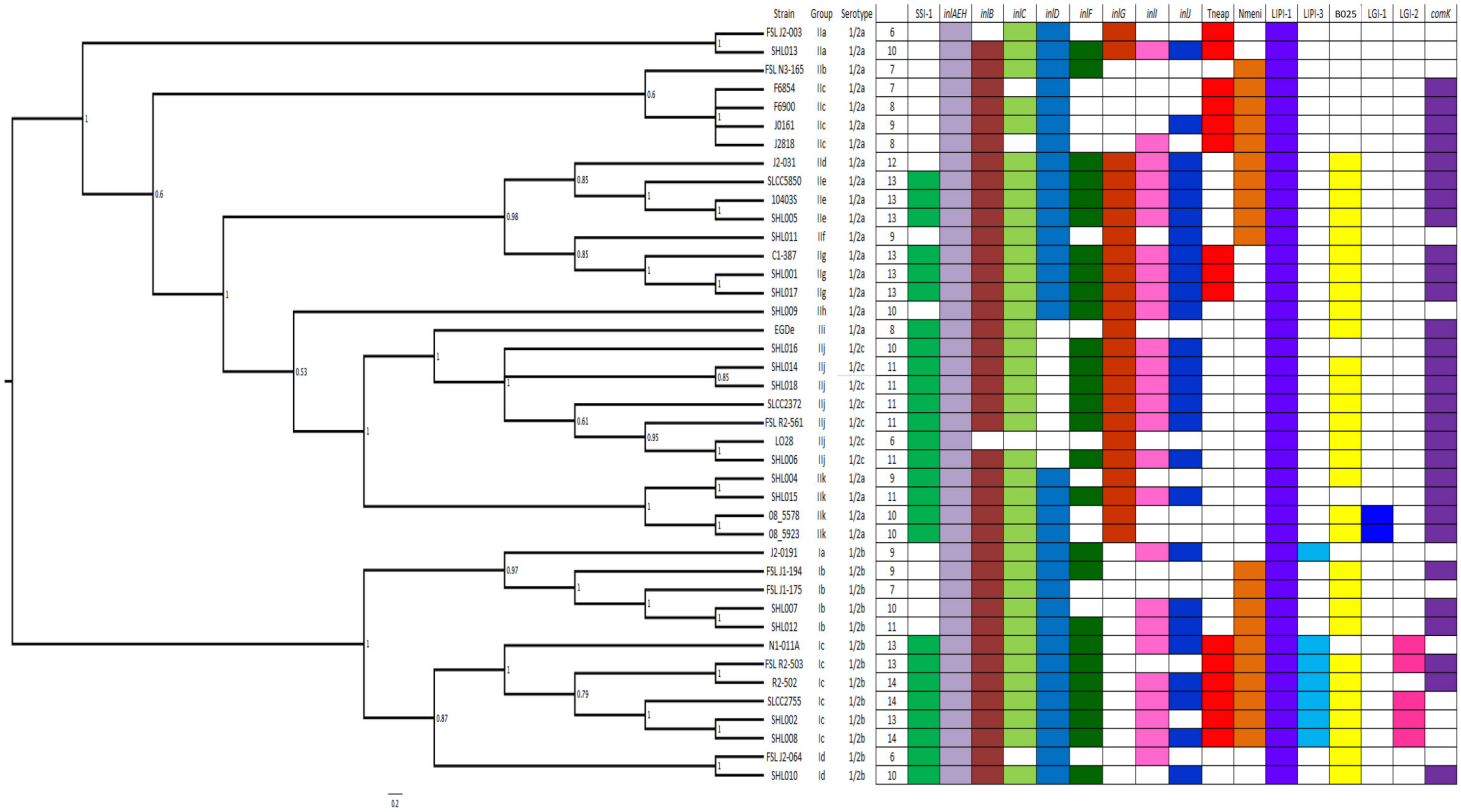
**Phylogenetic tree of 41 *L. monocytogenes* strains using whole genome sequencing data and distribution of important genetic elements**. More than 154,000 SNPs were used to construct the maximum likelihood phylogenetic tree for all compared genomes. The bootstrap value for each node was listed. The total number of present genetic elements examined was listed in the first column for each genome.

A maximum likelihood (ML) phylogenetic tree was constructed using more than 154,000 SNPs, which were identified from core genome alignments (Figure 1). The 41 *L. monocytogenes* genomes were divided into two lineages based on phylogenetic data. Serotype 1/2b strains belonged to lineage I, whereas serotypes 1/2a and 1/2c strains belonged to lineage II. These two lineages were further split into 4 (Ia to Id) and 11 (IIa to IIk) subgroups, respectively (Figure 1). The number of SNPs differences (standard deviation) between the four subgroups of lineage I ranged from 7593 (±47 SNPs) to 10,681 SNPs (±94 SNPs) (Table S1). The number of SNPs differences between lineage II subgroups were more than 18,692 SNPs (±86 SNPs) with one exception (Table S1). The SNPs differences between subgroups IIi (strain EGDe) and IIj (serotype 1/2c) were only 1112 SNPs (±30 SNPs). Serotype 1/2c strains originated from EGDe, which was the middle point between serotypes 1/2a and 1/2c. EGDe and all serotype 1/2 strains belonged to clonal complex (CC) 9.

In lineage I, some clinical strains were closely related each other (Figure 1, Table S2). Strain SHL007 (2011) displayed only 30 SNPs (±5 SNPs) differences from SHL012 (2010), which belonged to sequence type (ST) 87 same as reference genome FSL J1-175. The SNPs difference between SHL002 (2007) and SHL008 (2012) was 194 SNPs (±8 SNPs), and both strains belonged to ST3.

Lineage II strains displayed a divergent structure between subgroups (Table S1). Eight 1/2a strains from China were scattered in different subgroups with large SNPs differences. SHL013 recovered from an infant who died of listeriosis had more than 24,000 SNPs differences compared to other genomes except FSL J2-003. Another lethal strain, SHL009, also showed more than 20,000 SNPs differences compared to the rest genomes. Several foodborne and clinical strains were genetically closed. However, there was no epidemiological data available to make any foodborne illness connection. For example, the difference was 60 SNPs (±4 SNPs) between SHL001 (human) and SHL017 (bean), and only 11 SNPs (±2 SNPs) between SHL004 (human), and SHL015 (beef) (Table S2). Serotype 1/2c strains showed a clonal structure and the differences between the 1/2c genomes were no more than 300 SNPs (Table S2).

Based on SNPs difference, several strains from China appeared to have a close evolutionary relationship to those from North America. SHL007 to SHL008 had no more than 120 SNPs difference compared to FSLJ1-175 and R2-502 from the United States (Table S2). Clinical strain SHL004 displayed 94 SNPs (±7 SNPs) and 93 SNPs (±7 SNPs) differences compared to strains 08-5578 and 08-5923, respectively, which were recovered from a large foodborne outbreak in Canada in 2008 (Table S1). Similarly, SHL015 had only 97 SNPs (±7 SNPs) and 96 SNPs (±6 SNPs) differences compared to 08-5578 and 08-5923, respectively.

### Phylogenetic and comparative genomic analyses between strains from china (SHL004 and SHL015) and the outbreak strains (08-5578 and 08-5923) of canada

SHL004 and SHL015 were closely clustered with 08-5578 and 08-5923 in subgroup IIk (Figure 1). They belonged to CC8 that was identified as epidemic clone V (ECV). The SNPs differences between these genomes ranged from 11 to 97 SNPs (Table S2). Their genome organizations were displayed in Figure S1. We also identified plasmids sequences in SHL004 and SHL015 (Figure S2). Contig number 9 of SHL015 (86,633 bp, GC content: 37%, 92 ORFs) showed 99% identities and 100% cover compared to plasmid pLMR479a (86,652 bp, accession number: HG813248). Contig number 9 of SHL004 (79,013 bp, GC content: 36.7%, 82 ORFs) showed 100% identities compared to pLMR479a. Both plasmid sequences from SHL004 and SHL015 were also highly conserved compared to plasmid pLM5578 (77,054 bp, accession number: CP001603) from strain 08-5578.

**Figure 2:**
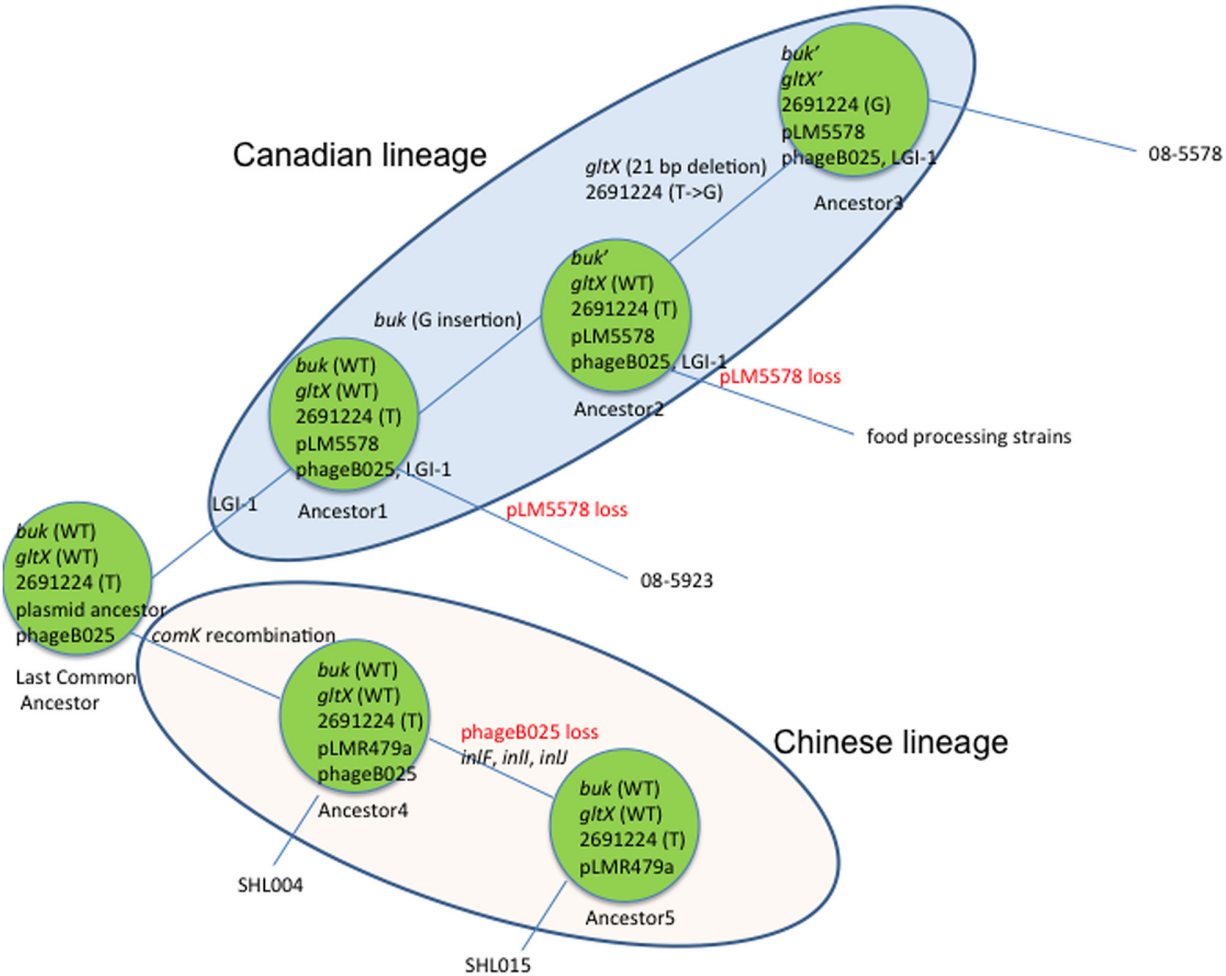
**Evolutionary model of subgroup IIk divided into Canadian and Chinese lineages**. The last common ancestor of subgroup IIk carrying plasmid pLM5578 was divided into two lineages. Both horizontal gene transfer and mutation play important roles for the divergence, such as the loss of plasmid and phage B025, and the acquisition of LGI-1.

There were certain variations in virulence determinants among the four genomes. SHL004 and SHL015 did not contain LGI-1 whereas 08-5578 and 08-5923 did. They contained one gene cluster encoding prophage proteins (phage B025) except SHL015. SHL015 carried genes *inlF, inlI*, and *inlJ*, which were absent in the other three strains. An evolutionary model for subgroup IIk strains was proposed (Figure 2), in which the last common ancestor containing plasmid was divided into the Chinese and Canadian lineages (Gilmour et al., 2010).

### Distribution of important genetic elements

Of 117 genes related to virulence, metabolism, and regulations determined (Table S3), 101 genes were present in all 41 genomes. We also found genes were present only in certain subgroups. For example, *lmo0150* (hypothetical protein) was present in subgroups IIa, IIi, IIj, and IIk; *lmo0471* (hypothetical protein) was found in subgroups IIi and IIj. Additionally, we identified strain specific genes within subgroups via comparing the genomes in the same subgroup (Table S4). The distribution of other genetic elements follows.

#### Stress survival islet (SSI-1)

SSI-1 was present in both lineages (Ic, Id, IIe, IIg, IIi, IIj, and IIk; Figure 1). The common ancestor of subgroups Ic and Id may have acquired SSI-1. The same happened to subgroups IIi, IIj, and IIk. In contrast, it seems that the ancestors of subgroups IIe and IIg obtained SSI-1 independently. A gene encoding transcriptional regulator was identified upstream of SSI-1. There was no other inserted sequence found in those without SSI-1 (Hein et al., 2011).

#### Internalins

The number of internalins in the genomes examined ranged from 5 (F6854 and LO28) to 10 (SHL013, SHL015, and subgroups IId, IIe, IIg, IIh; Figure 1). A gene cluster encoded four internalins including *inlG, inlH* (*inlC2*), *inlD*, and *inlE* (5′ to 3′). *InlH* and *inlE* were present in all genomes whereas *inlG* and *inlD* were found in different subgroups (Figure 1). *InlG* was present in the lineage II subgroups except IIb and IIc. However, lineage I strains did not contain *inlG*. Gene *inlD* was present in both lineages but subgroups IIi and IIj. SHL009 and SHL013 carried all four genes. Additionally, the presence of *inlC, inlF, inlI*, and *inlJ* were inconsistently scattered between different subgroups (Figure 1). SHL015 possessed all four internalins; however, SHL004 contained only *inlC*, which appeared to be lost independently four times on the phylogeny (Figure 1).

#### Non-coding RNAs (ncRNAs)

Among 150 ncRNAs (Table S5), 109 ncRNAs were present in all genomes with truncated lengths. For example, *rli91* in F6854 was 42 out of 88 bp. The rest ncRNAs remained in certain subgroups/strains (Table S5). *Rli29, rli85*, and *anti0469* were acquired by the common ancestor of subgroups IIi and IIj. *RliC* was identified in subgroups Id, IIi, and IIj. *Rli110* existed in all genomes except subgroup IIa genomes. *anti1457, anti1749, anti1758*, and *anti1974* were found in lineage II. *Rli28, rli50, rli78*, and *rli112* were identified in subgroups IIc, IIg, IIi, IIj, and IIk.

#### Secretion system proteins

Secretory pathway components of six identified secretion systems were present in all genomes except that certain genes were absent in some genomes (Table S6). The *tatA* gene was found exclusively in lineage II whereas *tatC* was present in lineage II and subgroup Id. *EsaC* was found in subgroups Ia, Ib, Id, IIi, and IIj. *SecE* was identified in all strains but F6854. *LspB* was only identified in EGDe.

#### Listeria pathogenicity islands (LIPIs), listeria genomic islands (LGIs), and prophage

The 41 genomes all contained LIPI-1 including *prfA, plcA, plcB, hly, mpl*, and *actA*. A 37-kb gene cluster encoding prophage proteins was located at the same location of LIPI-2 in *Listeria* (Figure 1, Table S7). This gene cluster was identified as phage B025 and contained no genes associated with virulence or antimicrobial resistance. B025 may have been acquired by the common ancestor of subgroups IId, IIe, IIf, and IIg. Independent loss events of B025 happened in subgroups IIj, and IIk (Figure 1). Moreover, LIPI-3 may have been independently obtained in subgroups Ia and Ic, encoding *llsG, llsH, llsX, llsB, llsY, llsD*, and *llsP* (Clayton et al., 2014).

LGI-1 exclusively existed in 08-5578 and 08-5923 in subgroup IIk. An insertion encoding 50 genes was identified at the same locus of LGI-1 in subgroup Ic except R2-502, termed as LGI-2 (Table S8). LIG-2 originated from phage and did not contain virulence genes based on the current annotation.

#### *ComK* prophage junction fragment

The *comK* phage insertions were present in 12 strains from China (Figure 1). These insertions carried divergent components with both indels and substitutions. Some strains in the same subgroup contained identical *comK* phage insertions, such as SHL001 and SHL017, SHL004, and SHL015. In contrast, subgroup Ib strains SHL007 and SHL012 carried different components in the *comK* junction fragment.

### CRISPR/cas system

Some *L. monocytogenes* strains from the same subgroup contained identical CRISPR spacer arrays, such as subgroup Id (Table S9). EGDe (1/2a, subgroup IIi) contained the same spacers as 1/2c strains (subgroup IIj). Several strains from China contained identical spacers that were different from those of the rest strains in the same subgroup, such as subgroup Ic strains SHL002 and SHL008 (Table S9). The number of spacers in different strains varied. There were 68 spacers in SHL001 and SHL017, but no spacer present in SHL009.

Two *cas* gene clusters were identified, Tneap and Nmeni (Figure 1). Tneap consisted of *cas6, cst1, cst2, cas5t, cas3, cas1*, and *cas2-1*, whereas Nmeni included *csn2, cas2-2, cas1*, and *csn1*. Subgroups Ic and Ic strains carried both *cas* clusters. In contrast, subgroups Ia, Id, IIh, IIi, IIj, and IIk did not contain any *cas* genes. The other subgroups contained either Tneap or Nmeni.

## Discussion

In the present study, we sequenced 17 *L. monocytogenes* strains recovered from humans and food in China and built a phylogenetic framework for *L. monocytogenes* using these genomes against publicly available data from diverse sources and locations. *L. monocytogenes* serotypes 1/2a and 1/2b displayed a divergent structure. In contrast, serotype 1/2c showed a clonal structure with small SNPs differences (Figure 1, Table S2). The strains from China contained extensive diversification, suggesting the evolutionary diversity and complex of *L. monocytogenes* in China.

Our findings provided detailed and comprehensive information on *L. monocytogenes* evolution and diversity. Four genomes belonging to CC8 were grouped together, SHL004 (human), SHL015 (beef), and two outbreak isolates 08-5578 and 08-5923 in Canada (Gilmour et al., 2010). Intriguingly, the isolation times for these two pair strains were close, September and August 2008, respectively. The proposed evolutionary model (Figure 2) suggests this *L. monocytogenes* clone complex may have circulated globally and the strains from different geographic locations have split into two subgroups.

Clinical strains of serotype 1/2b recovered from different years in Shanghai, China, (SHL002 in 2007 and SHL008 in 2012; SHL007 in 2011 and SHL012 in 2010) showed close genetic relatedness, suggesting these clones remain and circulate locally. SHL002 and SHL008 shared most recent common ancestor. SHL007 and SHL012 also originated from the same ancestor. Additionally, clinical strains of serotype 1/2a were grouped together with those from foods (SHL015 from beef and SHL017 from bean). Such an association indicated a possible foodborne transmission of listeriosis to humans.

The distribution of virulence factors indicated variations in virulence potentials and evolutionary histories in different subgroups. The mean numbers of virulence factors examined in subgroups Ic and IIg (Figure 1) were higher than other subgroups suggesting a link between these genetic factors and observed differences in pathogenicity, survival, and risk in causing diseases.

Internalins play essential roles in host-cell interactions of *L. monocytogenes* (Bierne et al., 2007) and the presence and distribution of internalins relate to differences in virulence potential (Rychli et al., 2014). InlC is essential in liver infection, cell-to-cell spread, and interactions with host cells (Leung et al., 2013). Since *inlD* is related to invasion activity (Seveau et al., 2007), serotype 1/2a and 1/2b strains are likely possess greater invasion ability than 1/2c strains. The distribution of internalins such as *inlD* and *inlG* suggests that formation of internalin clusters may be a multiple-step event and their acquisition could be related to divergence of these subgroups.

Genomic islands also play a vital role in survival and virulence of *L. monocytogenes* and their distribution appears to underpin different phenotypes observed in virulence among various subgroups. SSI-1, which was present in several subgroups (Ic, Id, IIe, IIg, IIi, IIj, and IIk), contributed to survival under low pH and high salt concentration (Ryan et al., 2010) and help *L. monocytogenes* to pass through the stomach and gut (Rychli et al., 2014). Moreover, LIPI-3 is likely to be acquired independently in subgroups Ia and Ic, known to encode hemolytic and cytotoxic factors as well as contributing to virulence of *L. monocytogenes* (Cotter et al., 2008). A total of 29 genomes including 12 from China contained *comK* phage junction fragment. It is noteworthy that the sequence variation in *comK* junction fragment may account for rapid adaptation and persistence in food processing plants and the contamination of RTE meats (Verghese et al., 2011).

The ncRNAs distribution also highlighted potential differences in *L. monocytogenes* pathogenesis (Mraheil et al., 2011). *rli29, rli85*, and *rliC* were present in EGDe (IIi) and 1/2c strains (IIj), all of which related to intracellular up-regulation in macrophage, although *rliC* was reported down-regulated in lineage III strains (Deng et al., 2010). These three ncRNAs are likely to be acquired by the common ancestor of subgroups IIi and IIj. Certain subgroups (IIc, IId, IIg, IIi, IIj, and IIk) carried *rli50*, documented previously as being involved in virulence in mice cells (Toledo-Arana et al., 2009; Mraheil et al., 2011).

In addition, strains in the same subgroups with small core SNP differences had different phenotypes in virulence due to possibly some strain specific genes (Table S4). SHL007 contained genes encoding hemolysins (locus tags: I615_15111 and I615_15116) compared to SHL012; SHL013 carried multidrug efflux protein (I622_01390), ABC transporter proteins (I622_03330 and I622_03805) and flagella proteins (I622_05059 and I622_05069) compared to FSL J2-003.

The absence of *cas* gene clusters was not uncommon in *L. monocytogenes* (Hain et al., 2012; Kuenne et al., 2013). This lack of enzyme encoding DNA in these strains would likely render the entire CRISPR system unfunctional, which could facilitate the strains in these subgroups in acquiring foreign genetic elements including antibiotic resistance genes (Palmer and Gilmore, 2010). As the difficulty of assembling *cas* genes region in our draft genome, the findings here may be inconsistent.

However, as we got draft genomes for all strains, the limitation for the current study includes sequence gap, sequence error, and could achieve better genome quality with alternative assembly methods.

In summary, *L. monocytogenes* strains from China displayed a divergent population structure. Links between clinical and food strains, and a possible connection of strains from China and those associated with outbreak in another country indicate that whole genome sequencing data provide valuable information in public health and basic research. The differences in the distribution of virulence factors among *L. monocytogenes* from various sources highlighted variations in pathogenicity and the importance of horizontal gene transfer in the evolution and divergence of *L. monocytogenes*. The great resolution and power of whole genome sequencing advances a better and deeper understanding of the origins, emergence, and relationship of the dangerous foodborne pathogens.

## Data deposition

This project has been deposited at the Nucleotide database at NCBI under the GenBank accession numbers listed in Table 2.

## Author contributions

Conceived and designed the experiments: JM, JZ, and GC. Performed the experiments: JZ, GC, XY, and HP. Contributed reagents/materials/analysis tools: XX, MA, EB, PL. Wrote the paper: JZ, GC.

## Funding

This study was supported in part by the National Science and Technology Key Project (No. 2012ZX10004215-003), China-U.S. Collaborative Program on Emerging and Re-emerging Infectious Diseases (1U2GGH000961-01&5U2GGH000961-02) and the Joint Institute for Food Safety & Applied Nutrition, University of Maryland.

### Conflict of interest statement

The authors declare that the research was conducted in the absence of any commercial or financial relationships that could be construed as a potential conflict of interest.

## Supplementary material

Figure S1 **Genetic organization of SHL004, SHL015, 08-5578, and 08-5923**. Strain 08-5578 was selected as reference sequence in the inner circle.

Figure S2 **Sequence comparison of plasmids pLMR479a, pLM5578, and those from strains SHL004 and SHL015**.

Table S1 **Pairwise distance matrix of SNP number differences with stand deviation for 11 subgroups of compared genomes**.

Table S2 **Pairwise distance matrix of SNP number differences with stand deviation for 41 compared genomes Strain**.

Table S3 **The distribution of 117 genes related to virulence, regulation, and metabolism**.

Table S4 **Strain specific genes identified from the comparison between strains with close relationship within the same subgroups**. Strain specific genes in SHL007 compared to SHL012 (subgroup Ib).

Table S5 **Distribution of ncRNAs in all compared genomes**.

Table S6 **Distribution of secrete systems proteins in all compared genomes**.

Table S7 **General characterizations of phage B025 using complete genome C1-387 as example**.

Table S8 **General characterizations of LGI-2 using complete genome SLCC2755 as example**.

Table S9 **CRISPR arrays sequence in compared genomes**.
