# Supplementary figures and images for: Evolution and Diversity of Listeria monocytogenes from Clinical and Food Samples in Shanghai, China
Source: Front Microbiol. 2016 Jul 22;7:1138. doi: 10.3389/fmicb.2016.01138 (PMC4956650; doi:10.3389/fmicb.2016.01138)

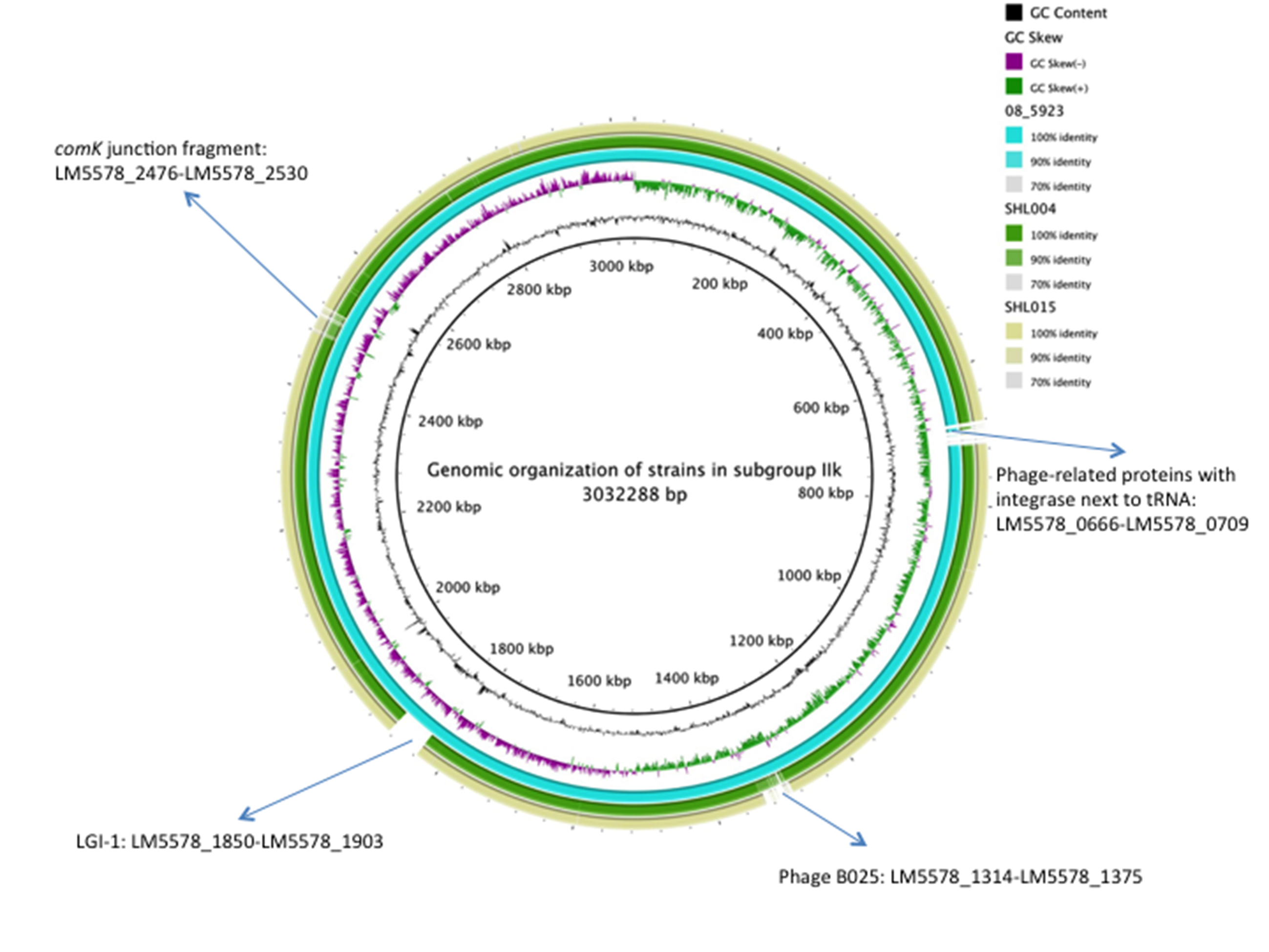

Supplement: Figure S1 — Genetic organization of SHL004, SHL015, 08-5578, and 08-5923. Strain 08-5578 was selected as reference sequence in the inner circle. [file Image1.TIF]

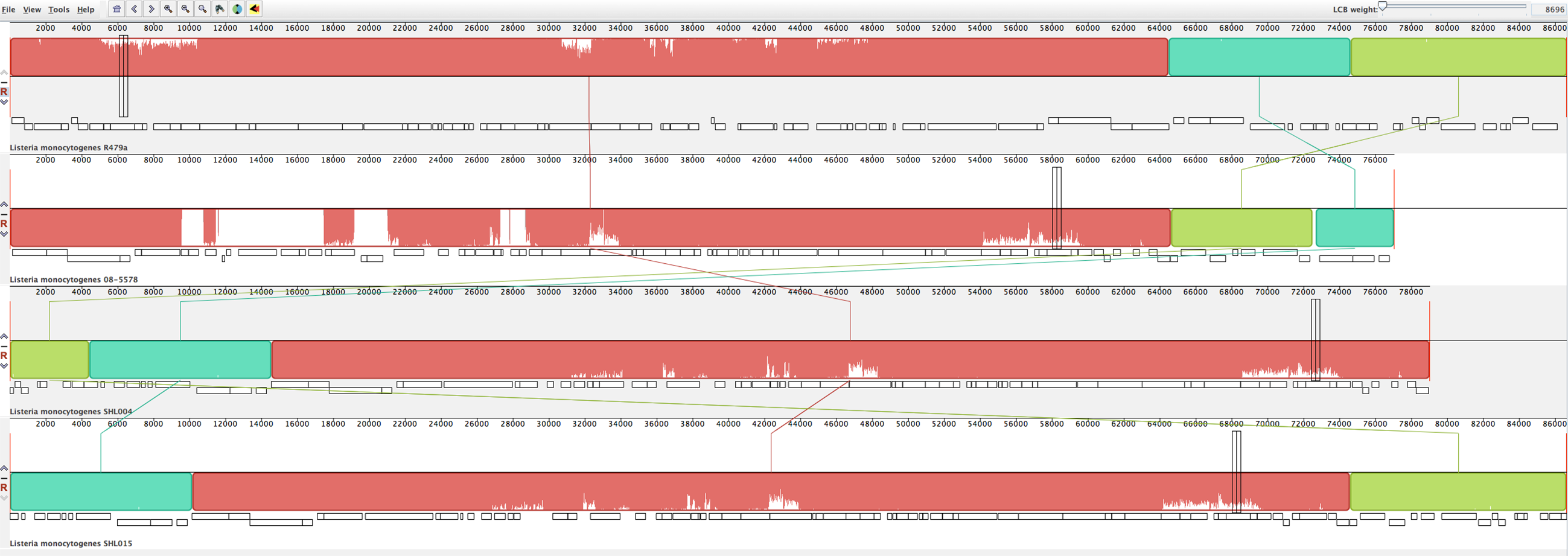

Supplement: Figure S2 — Sequence comparison of plasmids pLMR479a, pLM5578, and those from strains SHL004 and SHL015. [file Image2.TIF]
